# Psychometric Analysis and Effectiveness of the Psychological Readiness of Injured Athlete to Return to Sport (PRIA-RS) Questionnaire on Injured Soccer Players

**DOI:** 10.3390/ijerph17051536

**Published:** 2020-02-27

**Authors:** Pedro Gómez-Piqueras, Clare Ardern, Alejandro Prieto-Ayuso, Francisco Javier Robles-Palazón, Antonio Cejudo, Pilar Sainz de Baranda, Aurelio Olmedilla

**Affiliations:** 1 Lille LOSC FC, 59800 Lille, France; 2 Division of Physiotherapy, Linköping University, 581 83 Linköping, Sweden; c.Ardern@latrobe.edu.au; 3 Faculty of Education, University of Castilla-La Mancha, 16002 Cuenca, Spain; Alejandro.prieto@uclm.es; 4 Department of Physical Activity and Sport, Faculty of Sport Sciences, Campus of Excellence Mare Nostrum, University of Murcia, 30100 Murcia, Spain; franciscojavier.robles1@gmail.com (F.J.R.-P.); antonio.cejudo@um.es (A.C.); psainzdebaranda@um.es (P.S.d.B.); 5 Department of Personality, Evaluation and Psychological Treatment, Faculty of Psychology, University of Murcia, 30100 Murcia, Spain; olmedilla@um.es

**Keywords:** injury, psychology readiness, soccer, return to training

## Abstract

The decision-making process about when an athlete may safely return to training and competition after an injury is a difficult decision. Safe return to training and competition is characterised by physical and psychological readiness to return to the sport. The objectives of this study are (1) to assess the measurement properties of the Psychological Readiness of Injured Athlete to Return to Sport questionnaire (PRIA-RS), and (2) to analyse the effectiveness which the PRIA-RS questionnaire possesses when applied during four consecutive seasons on professional soccer players. One hundred and nine male soccer players from the Albacete Soccer Club (Spain) were involved during four consecutive seasons for the current study: 2012–2013, 2013–2014, 2014–2015 and 2015–2016. Psychometric analysis (validity, reliability, internal consistency and effectiveness) and external psychometric analysis (evaluating measures of patient-reported outcomes (EMPRO)) were confirmed and supported. The main results of the study reveal that the psychometric properties of this questionnaire are optimum for their application in a professional sports context.

## 1. Introduction

Sports injury is inherent to sports practice. The demands of some sports, such as soccer set athletes, have a higher risk of injury than other sports [1]. Indeed, in a recent meta-analysis, it has been found an injury incidence of 8.1 injuries/1000 hours of exposure in professional soccer players [2]. However, the importance of injuries in soccer is not only based on the primary event; up to 25–30% of total injuries have been categorised as re-injuries in previous research [3], and the residual effects derived from a secondary event seem to be worse than the primary one (e.g., longer absences [4]). Therefore, it is extremely necessary to improve the recovery process and return-to-sport (RTS) decision in order to reduce re-injuries in soccer.

Psychological responses, including stress, anxiety, depression, confidence, optimism, motivation, and the fear of re-injury, have an important impact on recovery from injury [5,6,7,8,9]. Psychological responses after injury differ according to personal factors (e.g., characteristics of the injury, personality, perceptions) and situational factors (e.g., type of sport, external support, rehabilitation environment) [10]. Besides, psychological responses change as the athlete progresses through the rehabilitation phase; there is often an increase in negative psychological responses close to the time the athlete is preparing to return to his or her sport [11,12,13], which might affect the quality of the athlete’s performance and increase the risk of re-injury [14]. Consequently, the decision-making process about when the athlete may safely return to the training and competition after injury [15] is key, and clinicians should consider screening athletes during the rehabilitation phase to identify those with potentially maladaptive psychological responses to injury [2]. During the last years, some guidelines have been developed in order to help clinicians and coaches make an informed decision as to whether an injured athlete may safely return to practice and/or competition [15]. Safe return to training and competition is characterised by physical and psychological readiness to return to sport [16].

Therefore, the Psychological Readiness of Injured Athlete to Return to Sport (PRIA-RS) questionnaire was developed to help practitioners assess a soccer player´s psychological readiness to return to soccer training after injury [17]. Although previous psychological scales with similar purposes have been published, such as Injury Psychological Readiness to Return to Sport (I-PRRS) [18], Re-Injury Anxiety Inventory (RIAI) [19] and Anterior Cruciate Ligament-Return to Sport after Injury (ACL-RSI) [20], they present some limitations for this population because they only assess one aspect of psychological readiness to return to sport (e.g., the I-PRRS comprises six questions, all regarding confidence; and the RIAI is composed of 28 items, all related to re-injury anxiety), or are injury-specific (e.g., the ACL-RSI was developed for athletes with anterior cruciate ligament injury). In this context, the PRIA-RS questionnaire is a useful tool that enables a comprehensive screening of psychological readiness to RTS based on the confidence, the individual perception, the insecurity and the fear of re-injury reported by the athlete at the end of the recovery process. The PRIA–RS questionnaire is comprised of 10 questions where each question is scored on a 5-point Likert scale (50-total points), with a higher score representing a more positive psychological response. 

The process of development of the PRIA-RS [17] and its translation and adaptation to the English context [21] has been previously published. However, no study (to the authors’ knowledge) has analysed the psychometric properties and the effectiveness of this tool to assess psychological readiness to return to sport. The objectives of this study are (1) to assess the measurement properties of the PRIA-RS, and (2) to analyse the effectiveness which the PRIA-RS questionnaire possesses when applied during four consecutive seasons on professional soccer players.

## 2. Materials and Methods

### 2.1. Participants

A total of one hundred and nine male soccer players participated in the current study throughout four consecutive seasons: 2012–2013 (N = 25), 2013–2014 (N = 27), 2014–2015 (N = 28), and 2015–2016 (N = 29). All the participants belonged to the first team of the Albacete Soccer Club (Spain), which competed in the first two seasons of the study period in the 2nd Division “B” League, and the second two seasons of the study period in the 2nd Division “A” League. According to the playing position, 14 players were goalkeepers, 35 defenders, 20 midfielders, 24 wingers and 16 forwards. The players had a mean age of 25.28 ± 4.3 years (range 18–36), mean height of 177.51 ± 6.3 cm (range 169–192), mean body mass of 73.23 ± 5.6 kg (range 67–84), mean body fat percentage of 9.95 ± 0.7 (range 8.9–11.1) (Faulkner Formula-4 folds [22]) and had been playing soccer for a mean 17.57 ± 6.3 years (range 9–26). Injury events were prospectively registered in all participants during the four competitive seasons. Players who did not suffer an injury event during the follow-up period or suffered a time-loss injury with <7 days of absence were excluded from the final analysis, as they did not complete any PRIA-RS questionnaire.

This study was conducted following the ethical recommendations from the Declaration of Helsinki and was approved by the Ethics Committee of Clinical Research of the University of Castilla-La Mancha and University of Murcia (ID: 1551/2017). Both the Club and the soccer players provided informed consent to participate in the study, and the researcher group guaranteed that the collected data would be treated in a confidential way. There were no under-age participants.

### 2.2. Instrument

Patient-reported outcomes (PROs) are usually defined as a “measurement of any aspect of a patient´s health status that comes directly from the patient (i.e., without the interpretation of the patient´s responses by a physician or anyone else)” [23]. The employed PRO was the Psychological Readiness of Injured Athlete to Return to Sport (PRIA-RS) questionnaire, designed by Gómez-Piqueras et al. [17]. We assessed content validity qualitatively, using a Delphi process with a group of international experts during the development of the questionnaire [17]. The expert group comprised 16 participants (14 men, 2 women), with professional experience in sports psychology (*n* = 10), sports science (*n* = 5) and sports medicine (*n* = 1) of 19 ± 9.3 years.

### 2.3. Measurement Properties

Extraction of the psychometric properties was based on the definition from the EMPRO tool (evaluating measures of patient-reported outcomes). The EMPRO is a valid and reliable tool for comparing the properties of generic and specific instruments used to measure PROs [24]. It is a tool designed by the Spanish Cooperative Investigation Network for Health Service Outcomes Research (Red-YRYSS) to facilitate the standardized evaluation of the quality of PRO instruments [24].

The EMPRO consists of 39 items organized into eight attributes: conceptual and measurement model (7 items), reliability (8 items), validity (6 items), responsiveness (3 items), interpretability (3 items), administration burden (7 items), alternative modes of administration (2 items), and cross-cultural and linguistic adaptations (3 items). Each item consisted of a statement together with a short text to help in its interpretation and application. Reviewers expressed their degree of agreement with the statement on an ordinal Likert-type response scale: “Strongly agree” (4), “Agree” (3), “Disagree” (2), and “Strongly disagree” (1) [24].

The PRIA-RS questionnaire was evaluated by two independent researchers based on the available evidence and following the EMPRO tool recommendations for use. Options of “no information” and “not applicable” boxes were available to reviewers on multiple EMPRO items when there was insufficient or inappropriate information. In case of discrepancies between the evaluators, they attempted to reach an agreement. If this agreement was impossible, the intervention of a third evaluator was necessary.

### 2.4. Procedure

We conducted training and match surveillance to document any injury that happened during the 4-season study period. Injury definition and characteristics were recorded in accordance with the international recommendations [25]. Re-injuries, defined as an injury of the same type and in the same zone in the two months following the athlete´s return to training sessions [25], were also recorded. 

All players who sustained an injury with time loss of ≥7 days completed individually the PRIA-RS questionnaire the previous day (at the end of the last rehabilitation session) to return to full soccer training. When a player suffered more than a single injury during the follow-up period (e.g., re-injuries), a different questionnaire was filled in for each injury event. For injuries with time loss of less than 7 days, we expected psychological responses to be less relevant to the return to sport [12], and PRIA-RS was not used in these cases.

The questionnaire was handed to the athlete by the physical trainer of the team. The trainer was available while the athlete completed the questionnaire to clarify the general questionnaire instructions and to ensure minimal environmental noise, optimum lighting conditions, and an absence of interruptions. The questionnaire took 2–3 minutes to complete in all cases.

### 2.5. Data Analysis

A descriptive analysis of overall injury events (≥7 days of absence) was carried out for new injuries (“non re-injury”) and recurrent injuries (“re-injury”) recorded during the study period, and the incidence and overall (training and competition) days of absence per season and player were calculated. We calculated the mean PRIA-RS total score and the mean scores for individual questions.

We compared demographic characteristics (age, height, weight, experience and absence days) and PRIA-RS questionnaire scores between players who sustained a re-injury and those who did not sustain a re-injury. We used the Kolmogorov–Smirnov (K–S) test to assess the distribution of the data (normally or not normally distributed). We assessed between group differences using independent samples *t*-tests or Mann–Whitney U-tests, as appropriate. We used a chi-squared test to compare the number of re-injuries between offensive and defensive players, and between joint/ligament injuries and muscle/tendon injuries.

#### 2.5.1. Psychometric Properties

Consistent with the EMPRO manual, attribute scores were calculated as the mean of the responses to all items for that attribute, with a linear transformation to obtain the scores on a scale from 0 (minimum) to 100 (maximum). EMPRO scores were considered reasonably adequate it the scored at least 50 points. This threshold was agreed on based on the global recommendations made by the reviewers in priors studies where EMPRO was implemented [24,26,27]. More concretely and in the same way as in an earlier study, if the overall EMPRO score was higher than 50, the instrument obtained the category of “recommended with provisos or alterations” and if the instrument scored higher than 50 on every dimension, the instrument was categorized as “strongly recommended” [26].

#### 2.5.2. Validity and Effectiveness

To assess construct validity, we examined convergent and divergent validity. For the convergent validity, we calculated the correlation (Pearson’s r) between each question and the total PRIA-RS score (question–total correlation) [28].

For divergent validity and the effectiveness of the questionnaire, we compared the scores from athletes who sustained a re-injury to those who had not sustained a re-injury. We assessed the effect size (Cohen’s d) to determine the magnitude of the differences: trivial (>0.2), small (>0.2–0.6), moderate (>0.6–1.2) and large (>1.2–2) [29]. We also calculated the relative risk (RR) for each question and the PRIA-RS total score.

To assess structural validity, we conducted an exploratory factorial analysis (EFA) and a confirmatory factor analysis (CFA) with IBM SPSS v.22 program. An estimation method of weighted least squares was used due to the distribution of the sample, with direct oblimin rotation due to the existence of oblique factors [30].

#### 2.5.3. Reliability

To assess internal consistency, we calculated Cronbach´s alpha. A higher Cronbach alpha statistic suggests greater homogeneity among the questions that comprise the instrument. Also, the composite reliability of the construct and the analysis of the variance extracted were calculated.

**Figure 1:**
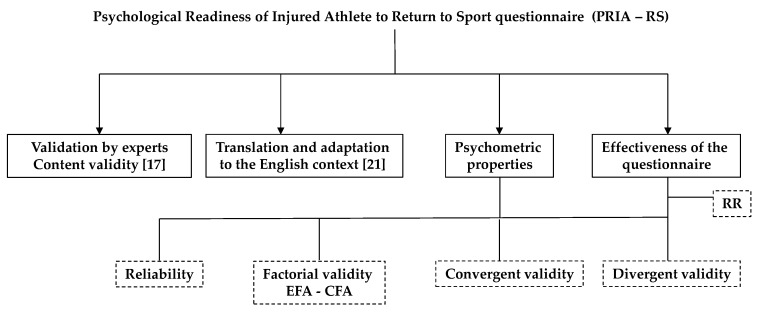
Study research design.

During the entire analysis process, we used the IBM SPSS v.22 (SPSS Inc, Chicago, IL, USA) program with a confidence level of 95%. Figure 1 represents the design of the study research.

## 3. Results

A total of 251 injuries in 102 players were recorded during the 4 seasons in which the study was carried out. However, of the 251 total injuries, only 111 (44.2%) possessed a severity of more than 7 days, which supposed an overall absence days from soccer practice of 2830 days. In 23 of these cases (20.7%), the soccer player suffered a re-injury of the same injury in the two months after his return to the training sessions. There were no differences in any of the recorded demographic variables (age, height, weight and experience) when dividing the sample into players who sustained a re-injury and those who did not. There were also no differences between both groups in relation to the absence days suffered, the type of injury and the predominant playing position (offensive or defensive; Table 1).

The individual question mean scores for the PRIA-RS ranged from 4.24 to 4.95 points (Table 2). The questions with the lowest mean score were those related to the perception of the physical condition (Question 3), the functional condition (Question 4), and the limitations or discomforts that the athletes felt about returning to the training (Question 5). The two questions with the highest mean score were the perception of the possibilities of the re-injury (Question 8) and perceived pressure to return to competition (Question 9).

### 3.1. Convergent Validity

Considering the rule of *Thumb* [28] for the interpretation of the size of the correlations, the question-total score correlation for questions 4, 5, 7 and 10 were high (r > 0.7), the question-total correlation for questions 3 and 6 were moderate (r > 0.5), and the question-total correlations for questions 1,2,8 and 9 were low (r < 0.5) (Table 3).

### 3.2. Divergent Validity

There were significant differences between the re-injury group and the non re-injury groups for 9 of the 10 PRIA-RS questions (Table 4). The effect sizes for the between-group differences were large (>1.2) for Questions 4, 5, 7, 10, and for the PRIA-RS total score (Table 4).

When categorizing the scores obtained by the athletes in two groups and seeking associations between a lower score and the appearance of a re-injury (x2), we found that they were significant for Questions 1, 4, 5, 6, 7,10 and for the total score (*p* < 0.05). In turn and as a measure of the permitted effectiveness [31], the relative risks (RR) were calculated to determine the association between the exposure (higher or lower score) and the event (re-injury or non re-injury). Only for Questions 4, 6, 7 and the total score was a significant risk detected (Table 5).

### 3.3. Structural Validity

The exploratory factor analysis indicated the existence of two factors that explained the 43.6% of the total variance (eigen values displayed in Table 6). This matrix shows that the first factor group Questions 1, 2, 4, 5, 7, 9 and 10, and the second factor group Questions 3, 6 and 8.

**Figure 2:**
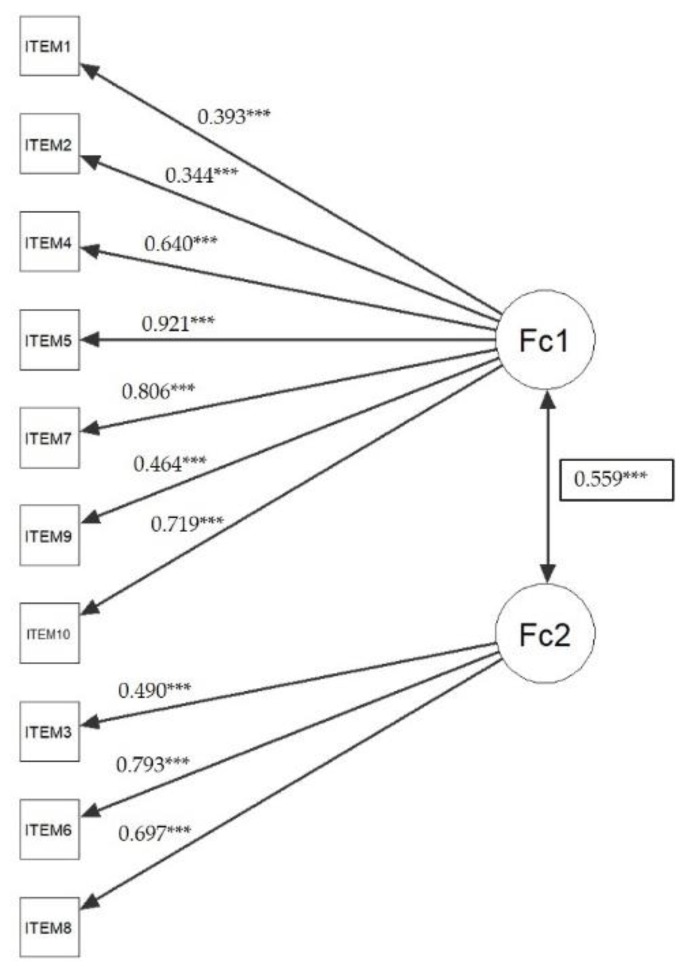
Confirmatory factor analysis. Chi-square = 57. 100 df. = 34, *p* = 0.008; CFI = 0.936; TLI = 0.915; SRMR = 0.0615; RMSEA = 0.0782 (lower CI = 0.0401; upper CI = 0.113). *** *p* < 0.001.

The exploratory confirmatory analysis in Figure 2 showed the convergent validity of the scale a CFA using the robust method of estimation “maximum likelihood” (ML). The model has a reasonably greater adjustment with respect to the RMSEA index with values below 0.08 [32], as illustrated in Figure 2. All the loadings for each item showed statistically significant differences, indicating that all the items evaluate the same construct, at least the standardized loads of six questions were close to or greater than 0.7 [33]. These findings confirm that the scale possesses convergent validity (Table S1). Discriminant validity was calculated by testing the variance extracted between the two factors. The square of the covariance between the factors obtained a value of 0.312, which was lower than the index of variance extracted from the first factor (0.416) and the second factor (0.451). These results provide the discriminant validity. 

### 3.4. Internal Consistency

The Cronbach´s alpha statistic for the PRIA-RS was 0.81, suggesting the questionnaire had high internal consistency (Table 7).

Additionally, the exploratory analysis of reliability was reckoned two more criteria to assess scale reliability: the composite reliability of the construct and the analysis of the variance extracted (Table 8). Table 8 shows acceptable levels in composite reliability “0.70” [34] and “0.5” in the analysis of the variance extracted [35].

### 3.5. External Psychometric Analysis

Finally, the previous psychometric analysis performed through the EMPRO tool by two external evaluators showed an overall score of 53.5 and a higher score than 50 in 4 of the 9 dimensions. Thus, and in accordance to the recommendations indicated above for EMPRO tool, the PRIA-RS questionnaire would be a psychometrically recommended PRO with provisos or alterations.

## 4. Discussion

Athletes often experience negative psychological responses when injured [6]. To assess the psychological readiness of the athlete, the days prior to his return to the training sessions are an important issue to obtain a secure RTS and with lower risk of re-injury. Several authors have indicated that the severity and the type of the injury could condition the psychological responses of the athlete [36]. In this study, no significant differences were found between the re-injury groups and non re-injury groups in relation to these and other demographic variables (age, height, weight, experience and playing position). Due to the complex [37] and multi-factor [38] nature of a sports injury, other variables should be analysed jointly with the psychological readiness at the time of determining the re-injury risk of an athlete.

Relevant information concerning the metric properties of PRIA-RS was obtained by two ways: external analysis (EMPRO) and analysis of the data obtained in 111 cases of sports injury.

On the one hand, few studies have used the EMPRO tool to evaluate the psychometric properties of questionnaires [26,27,39] in comparison with a most extended similar one like COSMIN checklist (consensus-based standards for the selection of health status measurement instruments) [40]. COSMIN and EMPRO both focus on PROs but with the EMPRO tool is possible to calculate an overall score per dimension or an overall score about the measurements properties. This is not possible with COSMIN and that is why EMPRO was chosen in the present study. Following the recommended values for this tool [18], the PRIA-RS questionnaire obtained a recommended psychometric evaluation (score >50), which invites us to use it.

On the other hand, when analysing the responses obtained in the 111 cases in which the PRIA-RS questionnaire was completed, we verified how the score was high for each one of the 10 questions which comprise it. On a 1–5 scale, we found that the average range of the score was situated between 4.24 and 4.95 points. The athlete’s desire to return to the sport as soon as possible could create an acquiescence effect [41], which could cause these high scores. This phenomenon should be considered and controlled [42].

Since the values of the psychological readiness were not obtained with scales different from the PRIA-RS, the convergent validity was analysed by means of the correlations which each of the questions presented with the final score of the questionnaire. The analysis showed that 6 of the 10 questions which were included in the questionnaire presented a moderate–high correlation with the final score of the same. Question 5 (feeling of discomfort or limitation) and Question 7 (security of the gesture) were the two questions which presented the highest correlation. In order for the convergent validity between the two scales, which measure the same construct to be optimum, correlation values are required between 0.40 and 0.80 [43]. All the questions of the PRIA-RS questionnaire presented a correlation within this range. Since preceding research studies are not known in which the correlation has been quantified between two scales of psychological readiness prior to RTS, it would be interesting in the same context to compare the results obtained with the PRIA-RS questionnaire and existing questionnaires more similar to it, such as the I-PRRS (Injury Psychological Readiness to Return to Sport) [9], the RIAI (Re-Injury Anxiety Inventory) [19] or the ACL-RSI (Anterior Cruciate Ligament-Return to Sport after Injury) [20].

In relation to the divergent validity of the questionnaire and in the same way as in the work by Webster et al. [20], it was analysed by comparing the scores obtained by the group of soccer players who returned to the training sessions with normality and the group that returned to the training sessions but who had a re-injury. For all the questions, except Question 8, the psychological readiness of the group without re-injury was higher than that of the group with re-injury. Although the appearance of an injury is a multi-factor phenomenon [38], this data could be indicating that the psychological component, as suggested in several studies [6,44,45,46,47], is a factor to be taken into account for a secure RTS. For Questions 4, 5, 7 and 10, the size of the effect of these differences was large. The perception about the functional condition, the discomfort, the security and overall condition seem to be the variables which best discriminate among the subjects with and without re-injury. Although the fear of re-injury has been one of the most studied psychological variables and related to an insecure return [5,8], in this study, no differences were detected between groups (Question 8). A greater acquiescence effect could be hypothesized for this question since any athlete could interpret that a non-optimum assessment in this questionnaire could slow down the decision from the coaching staff about his return to the training sessions.

The results of the exploratory factorial analysis (EFA) confirmed the structure of the questionnaire comprised by two main factors which explain 43.95% of the total variance. All the questions will be statistically located in one factor or the other. This data reaffirms the presence of all the questions within the questionnaire and that they are consistently adapted to the population of professional soccer players in the recovery phase of an injury. Likewise, the goodness of fit of the model has been verified by confirmatory factor analysis (CFA) through the exact (chi-square), comparative (CFI and TLI), and absolute adjustment (RMSEA and SRMR) indices. The results obtained in the PRIA-RS questionnaire have confirmed the good fit of the model, obtaining a non-significant chi-square (*p* < 0.05), values lower than 0.95 for the CFI (0.93) and TLI (0.91) indices, and close to 0.08 in the RMSEA (0.07) and SRMR (0.06). In addition, the relationship between both factors (0.55) has been observed, as well as the correlation between each factor with its items, being the 0.34 the lowest (item 1) and 0.92 the highest (item 5) values. All relationships presented statistically significant values.

The effectiveness of the PRIA-RS questionnaire, understood as its capacity to detect a return to sports with a greater risk of re-injury, were analysed by means of association tests between the obtained score and the appearance of the re-injury. In 3 of the 4 questions in which the RR can be statistically calculated (Questions 4, 6 and 7), we detected a significantly lower re-injury risk for the athletes who scored above 3. It is fitting to highlight that when studying this association for the total score of the questionnaire, we detected a “cut off” situated at 40 points. Based on the presented data, an athlete who obtained a score equal to or lower than 40 points in the questionnaire would present a re-injury risk which is 46.90 times greater than the player who scored above 40 points. Although the confidence interval for this value indicates a great variability in the data, this could indicate a possible trend [48]. In the wait for new studies in different contexts and sample sizes, the tendency of this data appears to indicate the optimum effectiveness of the PRIA-RS questionnaire at the time of predicting a re-injury, which highlights the importance of the psychological factor when implementing specific injury prevention programs [49].

Finally, we indicate that the internal consistency is a reliability measurement that represents the average of the correlations which are observed among the questions of a questionnaire. If the questions correlate with each other, it is understood that they quantify the same construct [30]. In normal conditions, it is expected that the consistency of a scale is between 0.70 and 0.90 [50]. The internal consistency of the PRIA-RS questionnaire was optimum since it presented a value of 0.81. A correlation higher than 0.90 would indicate that there is redundancy in the questions of the questionnaire and that several of them should be eliminated [50].

The current study presents a more thorough analysis (in comparison with previous psychometric research) of the validity and reliability of a questionnaire to assess the psychological readiness to return to sport. However, and despite the comprehensive analysis carried out, some limitations should be recognised. Firstly, the acquiescence and/or social desirability could have been one of the main potential sources of bias in the present self-reported tool; the injured player, who might know that the results of the questionnaire will determine his future return to competitive play, could slightly modify his responses with the aim of returning immediately to sports practice. Secondly, the severity of the participant’s injuries may also affect the results. The time of absence from the sport may influence the psychological readiness of players throughout rehabilitation. In this sense, the psychological readiness could also have been influenced by the player’s experience with previous similar injuries. Lastly, although this study was developed during four competitive seasons, the small sample size involved, belonging to a single professional Spanish soccer club, could restrict the generalization of the current results to other athlete cohorts and sport contexts. 

## 5. Conclusions

Having analysed the psychometric properties and the effectiveness of the PRIA-RS questionnaire, we can derive two main conclusions. First, the tool has demonstrated to be valid to determine the psychological readiness which a professional soccer player has on the day before the return to practice after his injury. Second, the use of the PRIA-RS questionnaire with soccer players could help to prevent the occurrence of a re-injury. Clinicians may, therefore, consider the use of this questionnaire for screening purposes during the rehabilitation phase in order to identify those athletes with potentially maladaptive psychological responses to injury. 

## Figures and Tables

**Table 1:** Comparison characteristics of re-injury group and non re-injury group.

	Re-Injury (*n* = 23)	Non Re-Injury (*n* = 88)	*p*	ES
Absence days ^2^	28.09 ± 28.32	24.82 ± 24.71	0.58	0.13
Age ^1^	24.83 ± 3.15	25.23 ± 3.36	0.60	0.12
Height (m) ^1^	1.76 ± 0.06	1.77 ± 0.05	0.24	0.18
Weight (kg) ^2^	74.30 ± 4.17	74.58 ± 3.67	0.91	0.07
Years of experience ^1^	17.78 ± 3.10	18.06 ± 3.33	0.72	0.08
Offensive players ^*^ Defensive players ^*^	11	37	0.61	chi-square
	12	51		
Joint/ligament injury Muscle/tendon injury	7	41	0.16	chi-square
	16	47		

^1^ Normal distribution. T, test. ^2^ Non-normal distribution. U, Mann–Whitney test. * Defensive playing position includes goalkeepers, defenders and midfielders; * offensive playing position includes wingers and forwards.

**Table 2:** Average and typical deviation of each of the questionnaire questions.

Question		M	SD
1	How do you evaluate the progression you have experienced during the rehabilitation/sport functional recovery period since your injury?	4.59	0.52
2	How is your mood?	4.75	0.43
3	What is your physical state in view of a potential return to the team?	4.33	0.66
4	How do you evaluate the functional status of your damaged area?	4.38	0.66
5	Do you feel any discomfort or limitations that prevent you from training as normal?	4.24	1.26
6	Are you feeling nervous about returning to regular training with the team?	4.82	0.63
7	How secure do you feel when performing physical actions or movements in the injured area?	4.40	0.75
8	Give an estimated percentage of how likely you are to experience a recurrence of the injury soon	4.95	0.22
9	What level of pressure do you feel in your surroundings to return to training with the team?	4.92	0.27
10	How would you evaluate your overall condition in view of a potential return to full training?	4.73	0.48

Response range (1–5). A higher score means better psychological predisposition in all the questions.

**Table 3:** Question–total correlations.

	Total Score	
	Coef.	Sig.
Question 1	0.47	0.00
Question 2	0.40	0.00
Question 3	0.51	0.00
Question 4	0.73	0.00
Question 5	0.87	0.00
Question 6	0.61	0.00
Question 7	0.80	0.00
Question 8	0.45	0.00
Question 9	0.46	0.00
Question 10	0.73	0.00

**Table 4:** Contrasts between scores of the re-injury group and the non re-injury group.

	Self-Perception Return to Sport			
	Re-Injury *n =* 23	Non Re-Injury *n* = 88	*p*	ES
Question 1	4.17 ± 0.57	4.70 ± 0.45	0.00	1.03
Question 2	4.35 ± 0.48	4.85 ± 0.35	0.00	1.19
Question 3	4.09 ± 0.73	4.40 ± 0.63	0.04	0.45
Question 4	3.61 ± 0.58	4.58 ± 0.51	0.00	1.77
Question 5	2.22 ± 0.99	4.77 ± 0.63	0.00	3.07
Question 6	4.30 ± 1.14	4.95 ± 0.30	0.00	0.77
Question 7	3.39 ± 0.58	4.66 ± 0.54	0.00	2.26
Question 8	4.91 ± 0.28	4.95 ± 0.20	0.43	0.16
Question 9	4.70 ± 0.47	4.98 ± 0.15	0.00	0.80
Question 10	4.13 ± 0.54	4.89 ± 0.31	0.00	1.72
TOTAL SCORE	39.78 ± 2.31	47.74 ± 2.33	0.00	3.43

*p*: *t*-test (normal distribution); ES: effect size Cohen´s *d.*

**Table 5:** Association between score and re-injury.

	Ranks	Re-Injury *n* = 23	Non Re-Injury *n* = 88	Chi-Square *x^2^*	RR (CI)
Question 1	≤3	2	0	0.00 *	-
	>3	21	88		
Question 2	≤3	0	0	-	-
	>3	23	88		
Question 3	≤3	5	7	0.058	3.21 (0.9–11.2)
	>3	18	81		
Question 4	≤3	10	1	0.00 *	66.92 (7.9–566.9) **
	>3	13	87		
Question 5	≤3	23	10	0.00*	-
	>3	0	78		
Question 6	≤3	7	2	0.00 *	18.8 (3.5–98.9) **
	>3	16	86		
Question 7	≤3	15	3	0.00 *	53.12 (12.6–223.3) **
	>3	8	85		
Question 8	>60%	0	0	-	-
	≤60%	23	88		
Question 9	≤3	0	0	-	-
	>3	23	88		
Question 10	≤3	1	0	0.04 *	-
	>3	22	88		
Total score	≤40	12	2	0.00 *	46.90 (9.2–237.7) **
	>40	11	86		

RR: relative risk; CI: confidence interval; -: statistical test not applicable; * *p* ≤ 0.05; ** significant relative risk (CI does not include 1).

**Table 6:** Rotated factor matrix.

Question	Factor 1	Factor 2
Question 1	0.41	
Question 2	0.44	
Question 3		0.46
Question 4	0.55	
Question 5	0.91	
Question 6		0.61
Question 7	0.67	
Question 8		0.81
Question 9	0.34	
Question 10	0.67	

**Table 7:** Item reliability statistics.

Question	M	Sd	Item–Rest Correlation	If Item Dropped Cronbach’s α
Question 1	3.41	0.529	0.374	0.801
Question 2	4.25	0.436	0.296	0.807
Question 3	3.67	0.665	0.378	0.802
Question 4	3.62	0.661	0.641	0.772
Question 5	1.76	1.266	0.755	0.770
Question 6	1.18	0.635	0.508	0.788
Question 7	3.60	0.754	0.719	0.759
Question 8	4.05	0.227	0.414	0.805
Question 9	4.08	0.274	0.412	0.803
Question 10	3.27	0.485	0.670	0.777

**Table 8:** Reliability of the measurement tool.

Question	Variance Extracted	Composite Reliability
Factor 1	0.401	0.810
Factor 2	0.609	0.647

## Supplementary Materials

The following are available online at https://www.mdpi.com/1660-4601/17/5/1536/s1.
