# Supplementary material for: Psychometric Analysis and Effectiveness of the Psychological Readiness of Injured Athlete to Return to Sport (PRIA-RS) Questionnaire on Injured Soccer Players
Source: Int J Environ Res Public Health. 2020 Feb 27;17(5):1536. doi: 10.3390/ijerph17051536 (PMC7084929; doi:10.3390/ijerph17051536)
Supplement: Supplementary file 1 [file ijerph-17-01536-s001.pdf]

Supplementary Table 1. Factor Loadings

				95% Confidence Interval				
**Factor**	**Indicator**	**Estimate**	**SE**	**Lower**	**Upper**	**Z**	**p**	**Stand. Estimate**
Factor 1	ITEM1	0.207	0.0503	0.1084	0.306	4.12	< .001	0.393
	ITEM2	0.149	0.0419	0.0671	0.231	3.56	< .001	0.344
	ITEM4	0.421	0.0587	0.3065	0.536	7.19	< .001	0.640
	ITEM5	1.161	0.0957	0.9736	1.349	12.13	< .001	0.921
	ITEM7	0.605	0.0608	0.4862	0.725	9.95	< .001	0.806
	ITEM9	0.127	0.0256	0.0765	0.177	4.95	< .001	0.464
	ITEM10	0.347	0.0411	0.2666	0.428	8.45	< .001	0.719
Factor 2	ITEM3	0.325	0.0688	0.1899	0.459	4.72	< .001	0.490
	ITEM6	0.502	0.0633	0.3776	0.626	7.93	< .001	0.793
	ITEM8	0.158	0.0224	0.1137	0.202	7.03	< .001	0.697
